# Rare Case of Posterior Reversible Leukoencephalopathy Syndrome Secondary to Acute Chest Syndrome

**DOI:** 10.1155/2016/4346953

**Published:** 2016-11-10

**Authors:** Rohit Aiyer, Daniel Klein, Yasir El-Sherif

**Affiliations:** ^1^Hofstra Northwell Health, Staten Island University Hospital, Department of Psychiatry, Staten Island, NY, USA; ^2^Hofstra Northwell Health, Staten Island University Hospital, Department of Radiology, Staten Island, NY, USA; ^3^Hofstra Northwell Health, Staten Island University Hospital, Department of Neurology, Staten Island, NY, USA

## Abstract

We present a case of 29/m with a history of sickle cell disease who presented to the emergency department with sudden onset of chest, trunk, extremity, and back pain, consistent in quality and severity with the patient's usual pain crises. Soon after admission to the medical unit for acute chest syndrome (ACS), the patient developed sudden onset of hypertension associated with left sided hemiplegia, lethargy, dysarthria, aphasia, and left sided facial droop. Neuroimaging revealed that on MRI Brain there was multifocal extensive signal abnormality and a small focal areas of hemorrhage compatible with posterior reversible leukoencephalopathy syndrome (PRES). Patient was treated with levetiracetam and phenytoin and improved soon afterwards, with resolution seen on follow-up MRI two months later.

## 1. Case

Patient is a 29/m, with a history of sickle cell disease and presenting to the emergency department with sudden onset of diffuse pain to chest, extremities, trunk, and back. This presentation in pain, with regards to quality and severity, was consistent with previous usual pain crises that the patient describes having in the past, and the patient was typically admitted to our inpatient medicine service for pain management with no current home medications. In the emergency department, the patient was given intravenous hydromorphone 4 mg for management of his pain. Patient's vital signs were within normal limits, including blood pressure of 113/64. Initial labs indicated hemoglobin of 8.0 and hematocrit of 21.1, as well as reticulocyte count of 9.58% and immature reticulocyte fraction of 42.2%, consistent with acute chest syndrome (ACS) secondary to sickle cell disease. All other laboratory findings were within normal range, including the patient's renal function (creatinine: 0.46 mg/dL, blood urea nitrogen: 9 mg/dL). Due to the patient's low hemoglobin levels, they were transfused 10 units of packed red blood cells before being admitted to the inpatient medical unit.

**Figure 1:**
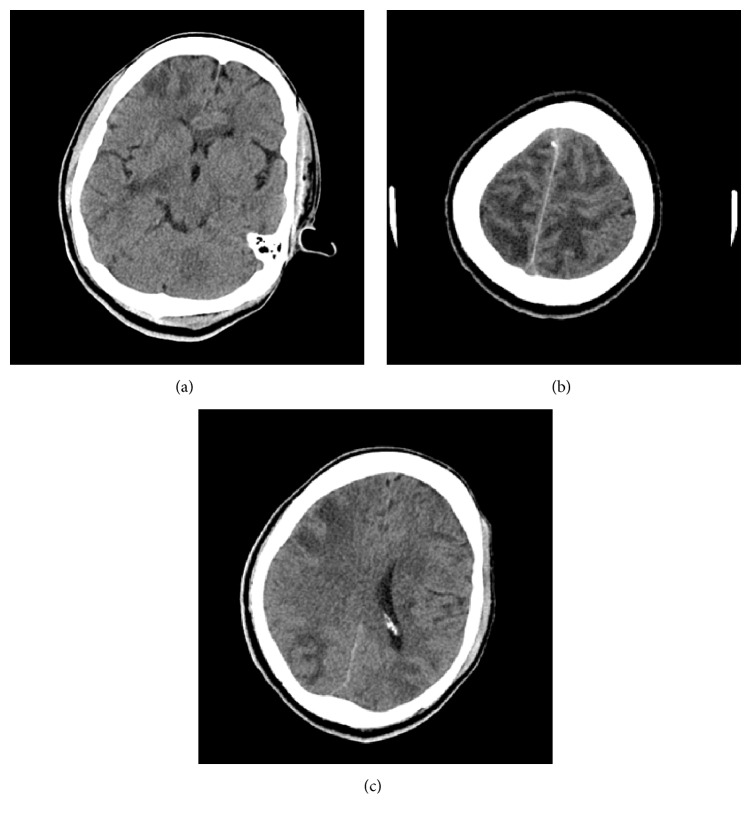
CT Head-Axial images demonstrate extensive multifocal areas of white matter hypoattenuation/edema.

While on the inpatient medical floor, patient's pain was managed by the pain management team. After two weeks, the patient had a sudden onset of hypertension secondary to acute kidney injury (creatinine: 5.19 mg/dL, blood urine nitrogen: 57 mg/dL), with a blood pressure reading of 212/96, and was found to have witnessed generalized tonic-clonic seizure lasting 1 minute. After the seizure, patient was found to have on examination, left facial droop, left upper extremity power of 3/5, left lower extremity power of 3/5, sensory neglect, dysarthria, perservation, and confusion. Based on neurological clinical findings, the weakness was likely due to a right focal onset with secondary generalization. Left hemiparesis was clearly present and was initially suspected to be due to an infarct. After Computed Tomography (CT) head imaging was performed it was reported as abnormal signal intensity in periventricular white matter, subcortical internal and external capsules, basal ganglia, and corpus callosum suspicious for posterior reversible encephalopathy syndrome (PRES) (Figures 1(a), 1(b), and 1(c)).

**Figure 2:**
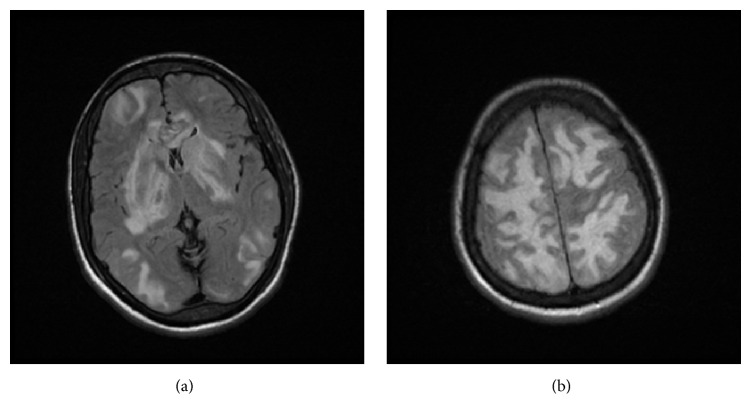
MRI Brain-Axial FLAIR-weighted images demonstrate the multifocal areas of edema involving the subcortical and deep white matter as well as the deep grey structures. Edema is also noted in the genu of the corpus collosum.

**Figure 3:**
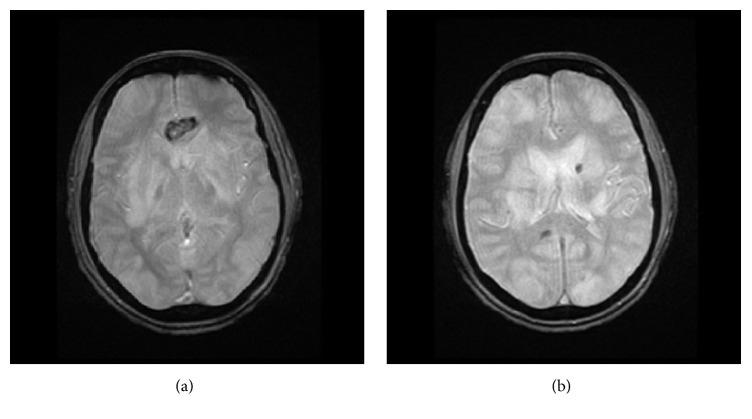
MRI Brain-Axial gradient echo-weighted images demonstrate susceptibility effect in the genu and splenium of the corpus collosum as well as the left frontal periventricular white matter compatible with multifocal hemorrhage.

These findings prompted further neuroradiological imaging. Subsequently, magnetic resonance imaging (MRI* GE*, 3 Tesla) of the brain, with and without gadolinium (DTPA), was ordered. MRI indicated that the patient had extensive white matter signal abnormality within the bilateral frontal and parietal lobes with extension into the temporal lobes supporting and initial impression of PRES (Figures 2(a), 2(b), 3(a), and 3(b)). As a result, patient was started on levetiracetam 500 milligrams intravenous q12h and phenytoin 100 milligrams intravenous q8h and placed on a video electroencephalogram (vEEG). The vEEG demonstrated focal slowing in right hemisphere, suggestive of underlying structural lesion. After a few days, the patient's symptoms resolved and became neurologically stable, and the patient was discharged on oral preparation of phenytoin 100 mg q8h and levetiracetam 500 milligrams q12h. After 8 weeks, the patient was tapered off phenytoin, and after 8 months the patient continues to be treated at the same dose of oral levetiracetam.

**Figure 4:**
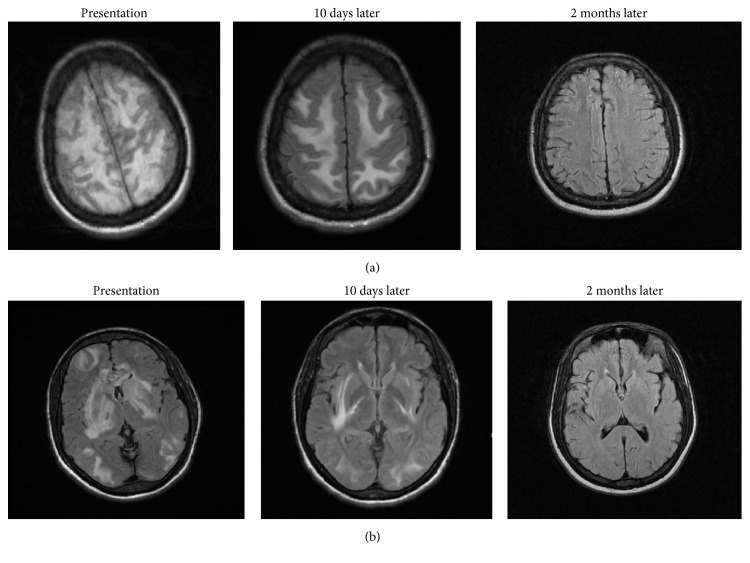
MRI Brain-Axial FLAIR-weighted images demonstrate improvement in the areas of edema over 10 days and 2 months.

Follow-up MRI Brain was done 10 days later, which showed partial resolution of the abnormal signal within the periventricular white matter, subcortical white matter, basal ganglia, and brainstem (Figures 4(a) and 4(b)). A further follow-up MRI was done 2 months later, which showed near complete resolution of hemorrhage and white matter abnormalities, as well as a completely normal neurological exam (Figures 4(a) and 4(b)).

## 2. Discussion

Posterior reversible leukoencephalopathy syndrome (PRES) was described by Hinchey et al. as a syndrome consistent with neurologic symptoms including headache, visual changes, seizures, or altered consciousness. Neuroimaging can confirm PRES, with MRI illustrating subcortical edema in the posterior regions of the cerebral hemispheres without infarction [1]. The risk factors for PRES can include the consumption of cytotoxic and immunosuppressive drugs, hypertensive encephalopathy, and eclampsia. Lesions in PRES are thought to be secondary to vasogenic edema that occurs mainly in the posterior cerebral hemispheres [2].

The pathophysiology of PRES is still relatively unknown with different perspectives. One hypothesis is that severe hypertension causes impaired cerebrovascular autoregulation, vasodilatation, and vasogenic edema [3]. Literature reviews on PRES and hypertension widely discuss the “endothelial hypothesis” as the pathophysiological cause for a patient's high blood pressure [4]. This hypothesis is based on the fact that endothelial dysfunction is due to insufficient production of nitric oxide (a potent vasodilator). In addition, there is endothelial activation which causes an increase in cell adhesion and narrowing of vessel lumen, which also contributes to hypertension [4]. These endothelial injuries cause for a narrowing of blood vessels and therefore create difficult in the flow of erythrocytes, leading to mechanical stress and resulting in many of the clinical signs and symptoms of PRES [4].

Another proposed idea is that toxicity of immunosuppressive drugs can lead to impaired endothelial homeostasis. However, PRES appears to be related to endothelial dysfunction that leads to cerebrovascular autoregulation impairment and vasogenic edema. Evidently, since endothelial dysfunction is thought to play a part in the cerebrovascular disease observed in Sickle Cell (SC) anemia, SC patients may have an increased risk of developing PRES [3].

Although seizures are the most common presentation, such as in our case presentation, PRES can also present with headache, visual disturbances, altered mental status, vomiting, ataxia, aphasia, or hemiparesis [3]. PRES is characterized by reversible radiographic signs of posterior leukoencephalopathy in the presence of headache, altered level of consciousness, and seizures with or without visual disturbances [5].

Our literature review showed that there are only 15 patients from the pediatric population (<18 years), and even less from the adult population (two patients). Therefore, to our knowledge, we believe that our case is only the third reported case in literature in an adult with PRES secondary to sickle cell crisis [6]. It should be noted that cases reported as stroke in the literature were unrecognized PRES, and therefore a literature search would overlook these findings [3]. Investigating the difference between PRES from a stroke can be difficult, yet the distinction is important as cerebral infarction suggests irreversible tissue injury. However, vasogenic edema and reversibility are thought to be the core sign of PRES [7].
